# Spatial Dependence of DNA Damage in Bacteria due to Low-Temperature Plasma Application as Assessed at the Single Cell Level

**DOI:** 10.1038/srep35646

**Published:** 2016-10-19

**Authors:** Angela Privat-Maldonado, Deborah O’Connell, Emma Welch, Roddy Vann, Marjan W. van der Woude

**Affiliations:** 1 Centre for Immunology and Infection, Department of Biology and Hull York Medical School, University of York, York, U.K; 2 York Plasma Institute, Department of Physics, University of York, York, U.K

## Abstract

Low temperature plasmas (LTPs) generate a cocktail of reactive nitrogen and oxygen species (RNOS) with bactericidal activity. The RNOS however are spatially unevenly distributed in the plasma. Here we test the hypothesis that this distribution will affect the mechanisms underpinning plasma bactericidal activity focussing on the level of DNA damage *in situ*. For the first time, a quantitative, single cell approach was applied to assess the level of DNA damage in bacteria as a function of the radial distance from the centre of the plasma jet. *Salmonella enterica* on a solid, dry surface was treated with two types of LTP: an atmospheric-pressure dielectric barrier discharge plasma jet (charged and neutral species) and a radio-frequency atmospheric-pressure plasma jet (neutral species). In both cases, there was an inverse correlation between the degree of DNA damage and the radial distance from the centre of the plasma, with the highest DNA damage occurring directly under the plasma. This trend was also observed with *Staphylococcus aureus*. LTP-generated UV radiation was eliminated as a contributing factor. Thus valuable mechanistic information can be obtained from assays on biological material, which can inform the development of LTP as a complementary or alternative therapy for (topical) bacterial infections.

Infections due to antimicrobial resistant pathogens continue to be a significant concern globally^1^. These lead to failure of therapies, and pose additional challenges for the development of new antimicrobial therapies. Hospital-acquired bacterial infections are one of the main nosocomial causes of morbidity, with a significant contribution from wound and surgical site infections^2^. In this context, there is much interest in identifying and developing new antibacterial therapies that could be applied topically. One approach arising from the field of physics is the use of atmospheric-pressure low-temperature plasmas (LTPs) as bactericidal agents^3^.

Plasma is the fourth state of matter and it can be generated by coupling sufficient quantities of energy to a gas to induce ionization. Plasmas that can deliver antimicrobial agents and at the same time operate below the thermal tissue damage threshold (<43 °C), the LTPs, are of particular interest for developing alternative or complementary antimicrobial therapies. LTPs are weakly ionised gases and consist of charged particles such as ions and electrons, neutrals, UV radiation and electromagnetic fields^4^. LTPs generated with molecular gases or sustained in ambient air contain highly reactive nitrogen and oxygen species (RNOS). These RNOS have been suggested to be the main agents responsible of the antimicrobial activity of LTPs^4^. RNOS can directly and indirectly affect the structure and function of proteins, lipids and nucleic acids^5^, and have been implicated in the mechanisms of cell death upon exposure to specific antibiotics^6^. UV photons produced in the plasma may also contribute to the bactericidal effect. They can damage DNA^7^ and may also photodissociate molecules to generate additional RNOS^8^.

Integrity of DNA is essential for survival, and thus the effect of LTPs on DNA has been a focus of interest. Reactive oxygen species produced in LTPs have been implicated in causing DNA break in purified DNA^9^,^10^. However, DNA in cells will benefit from the protection conferred by the membrane and cytoplasm, and damage can be repaired. Yet, with sufficient plasma exposure DNA damage can be induced in living cells, as shown for cells in liquid culture^11^.

Analysis of the components in the plasma phase has shown that specific reactive species produced in plasmas are spatially distributed, with most insight being available on reactive oxygen species^12^,^13^. Little is known about the effect of such distribution on the bactericidal action of LTPs as most analyses of biological effects are carried out at the population level, which leads to a loss of information on spatial variation. In the few studies where spatial information was retained, it was shown that spatial distribution of RNOS in the plasma correlated to the level of plasma induced breaks on purified DNA^14^,^15^.

Thus, distribution of RNOS in the plasma may affect both its efficacy and mechanism of bactericidal activity in a spatiotemporal manner. Understanding the radial variations of RNOS concentrations in the plasma jet and how this may affect the outcome of the bactericidal treatment is key for the development of effective therapeutic approaches. Here we begin to address this by analyzing the plasma induced double-stranded DNA (dsDNA) breaks in single bacteria, specifically as a function of the radial distance of the cell’s location to the centre of the plasma jet.

Here we compare the effects of two different LTP sources: an atmospheric-pressure dielectric barrier discharge (AP-DBD) jet, delivering charged and neutral species, and a radio-frequency capacitively-coupled plasma jet, known as the micro atmospheric-pressure plasma jet (μAPPJ), delivering mostly neutral species^13^,^16^. Bacteria on a solid, dry surface were exposed to plasma, making it possible to retain spatial information regarding effects of the treatment and to avoid complexities resulting from the chemical reactions between plasma and liquids. The development of a quantitative assay to analyse dsDNA breaks at the single cell level, allowed us to show that the level of dsDNA breaks induced in living cells by both types of LTPs shows a radial dependence. Furthermore, our data indicate this is due to RNOS distribution. We suggest this radial effect should be taken into consideration when developing LTPs as new antibacterial therapy for topical applications.

## Methods

### Bacterial strain and growth conditions

*Salmonella enterica* serovar Typhimurium ST4/74 and *Staphylococcus aureus* were grown aerobically at 37 °C in LB-Lennox (10 g tryptone, 5 g NaCl and 5 g yeast extract per litre) or agar (LB plus 15 g/L of agar), as required. LTP-treated cells were in the (late) logarithmic growth phase.

### Atmospheric-pressure dielectric barrier discharge (AP-DBD) jet

**Figure 1:**
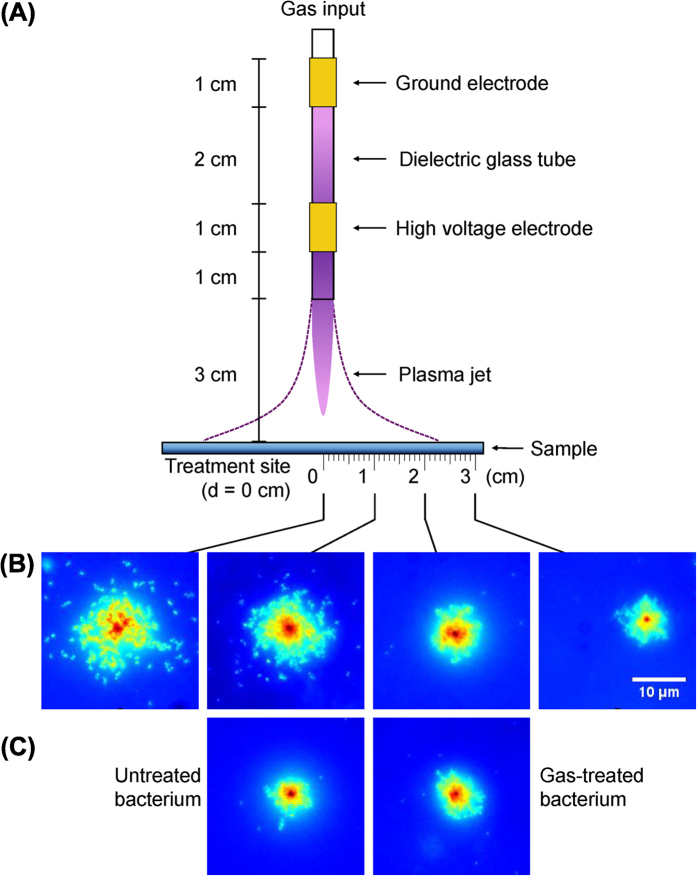
Schematic of the AP-DBD plasma jet and the effect of plasma treatment on bacterial DNA integrity. (**A**) Experimental arrangement for the AP-DBD plasma jet. Representative false colour images of bacterial DNA of (**B**) plasma-treated *S.* Typhimurium located at 0, 1, 2 and 3 cm from the treatment site (d = 0 cm), (**C**) untreated and gas-treated *S.* Typhimurium. Data were obtained from the modified DDD Assay as described (Methods). The level of dsDNA breaks derives from the area occupied by the fluorescently stained dsDNA fragments and varies as a function of the distance to the treatment site. See methods for details. Scale bar 10 μm.

An AP-DBD plasma jet designed and built in-house was used. The AP-DBD plasma jet is a linear-field device in which the gas flow and electric field are parallel^17^. The plasma jet is sustained through a streamer-like mechanism^18^. An oscilloscope (Agilent Technologies DSOX 2004A) with a signal generator function feeds a power amplifier with a sinusoidal voltage of 1Vpp with a frequency of approximately 10 kHz. The output of the amplifier (up to 60Vpp) feeds into the primary coil of a frequency-dependent transformer (Plasma Technics Inc.) installed in a polycarbonate high voltage enclosure where the plasma is generated. The transformer sends the voltage to the high voltage electrode with amplitudes between 5 and 12 kV (± 0.1; peak-to-peak), which was measured with a high voltage probe (Tektronix P6015A). The current was measured with a current monitor probe (Ion Physics Corporation) (see Supplementary Fig. S1). The plasma is generated in a hollow dielectric glass tube of 1 mm inner diameter and 6 mm outer diameter. The high voltage electrode is placed 10 mm above the nozzle of the glass tube (Fig. 1A). The ground electrode is located 20 mm upstream of the active electrode. The feed gas was controlled by mass flow controllers (Brooks; GFC) at 2 standard litres per minute (slm) in the axial direction.

**Figure 2:**
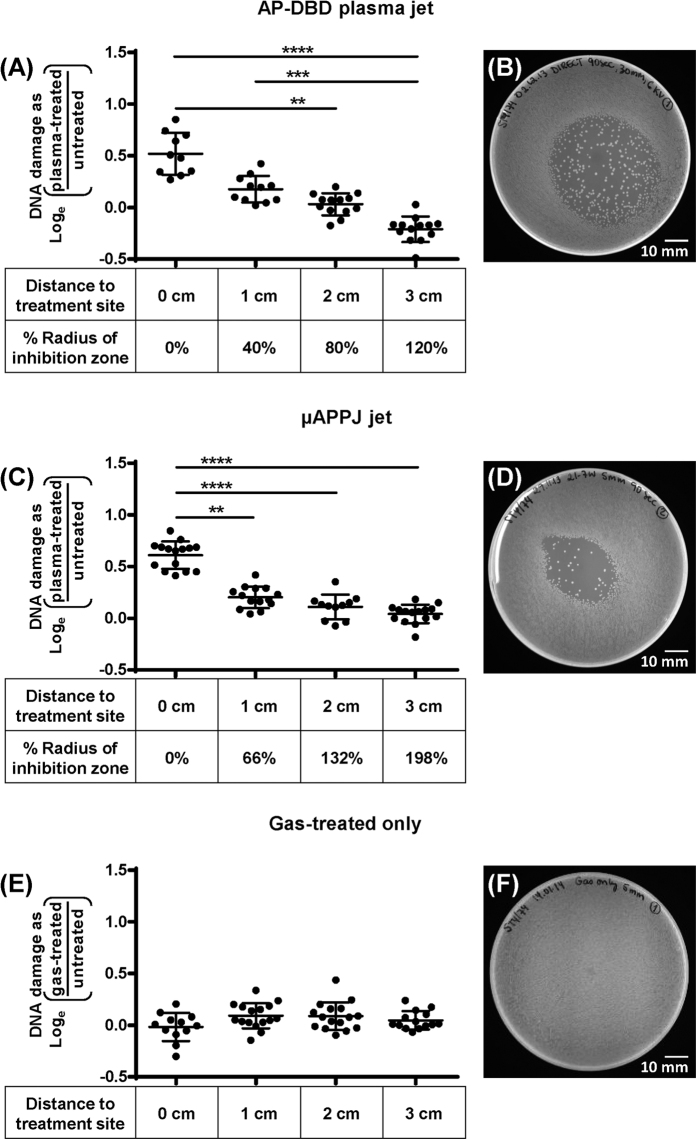
Plasma-dependent dsDNA break evidenced a radial dependence. After plasma exposure of *S.* Typhimurium, dsDNA breaks at the single cell level were assessed with the DDD Assay (**A,C,E**) and growth inhibition on LB agar plates was identified (**B,D,F**). Plots show DNA damage on plasma-treated bacteria at the single cell level as a function of their position to the treatment site (d = 0 cm) from *S.* Typhimurium exposed to (**A**) the AP-DBD plasma jet; (**C**) the μAPPJ jet and (**E**) gas only delivered by the AP-DBD setup at 5 mm from nozzle. Each dot represents a single cell; horizontal bars: mean values ± S.D.; ****P < 0.0001; ***P < 0.001; **P < 0.01. Ratio expressed as log_e_ ((radius plasma-treated cells)/(mean radius untreated cells)). The residual growth within the inhibition zone remained constant, i.e., no gradient of change was observed on LB agar plates with *S.* Typhimurium exposed to (**B**) AP-DBD plasma jet and (**D**) μAPPJ. (**F**) Bacteria in LB plates exposed to gas only delivered by the AP-DBD setup at 5 mm from nozzle. Representative plates for each treatment are shown. Scale bar 10 mm.

**Figure 3:**
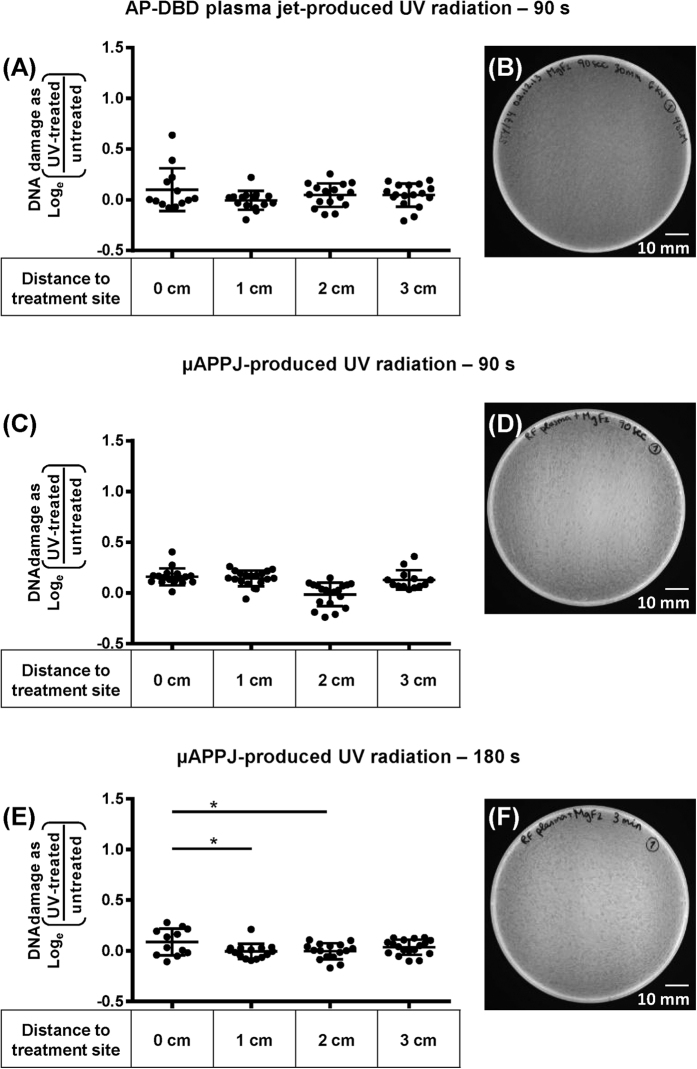
Plasma generated UV radiation induced negligible levels of DNA damage in S. Typhimurium and did not inhibit bacterial growth. dsDNA breaks at the single cell level were assessed with the DDD Assay (**A,C,E**) and growth inhibition on LB agar plates was tested (**B,D,F**) upon exposure to LTP-generated UV radiation above 121 nm. Radii of dispersion of bacterial DNA are shown for *S.* Typhimurium exposed to plasma-generated UV radiation in a RNOS-free environment by (**A**) the AP-DBD (90 seconds); (**C**) μAPPJ (90 seconds) and (**E**) μAPPJ plasma (180 seconds). Each dot represents a single cell; horizontal bars: mean values ± S.D. *P < 0.05. Ratio expressed as Log_e_ ((radius treated cells)/(mean radius untreated cells)). Representative plates for the corresponding treatment of *S.* Typhimurium with UV radiation generated by the AP-DBD plasma jet (**B**) and μAPPJ (**D,F**) in a RNOS-free environment are shown. Scale bar 10 mm.

Experimental conditions were standardized (see Results) with 30 mm distance to sample, 90 seconds exposure and feed gas consisting of He (>99.996% purity, BOC) with 0.5 vol% O_2_ (99.6% purity, BOC) delivered at 2 slm. The plasma was generated at 6 kV (peak-to-peak), 123 kHz for data shown in Figs 1, 2 and 3 and Supplementary Figs S3, S5 and S7, or at 12 kV and 31 kHz for data shown in Supplementary Figs S1, S2 and S8. Ambient humidity was between 20–40%. An optical emission spectrum is provided in Supplementary Fig. S2.

The macroscopic temperature of this plasma jet in the visible fraction (at 21 mm from the nozzle in the axial centre) was 74 +/−14 °C (see Supplementary Fig. S3). This was measured by optical emission spectroscopy using the second positive system of the nitrogen spectrum (HR4000 Fiber Optic Spectrometers). Data were acquired with SpectraSuite software (Ocean Optics) and analysed with an algorithm implemented in LabVIEW as described^19^. The temperature at the surface of the sample at 30 mm from the nozzle was measured with a thermocouple (Tenma) (see Results, Supplementary Fig. S3).

### Micro atmospheric-pressure plasma jet (μAPPJ)

The μAPPJ design is based on the COST reference microplasma jet^20^. Detailed characterisation of the plasma both experimentally, and through simulations and models, has been carried out. This includes a description of the plasma electron properties and measurements of reactive species e.g. atomic oxygen, atomic nitrogen, singlet delta oxygen, ozone and helium metastables^16^,^21^,^22^,^23^. Plasma was generated in a channel created by two plane parallel stainless steel electrodes mounted in between two quartz windows^24^. The charged particles were confined within the electrode gap inside the core plasma region and as a result neutral species and UV radiation dominate the effluent characteristics^9^. The temperature of the plasma was below 35 °C at distances greater than 5 mm from the exit in the axial direction^25^.

For μAPPJ application the distance to the sample was standardized at 5 mm (see Results). The length of treatment, gas composition and flow rate were the same as described above for the AP-DBD plasma jet. Ambient humidity was in the range of 20–30%. Plasma was generated at 13.56 MHz. The subtractive method was used to calculate the discharge power of the μAPPJ^26^,^27^. The coupled power and input current were measured with (plasma on) and without plasma (plasma off) using the Vigilant Power Monitor (SOLAYL SAS). The difference between both curves was used as an approximation of the power dissipated in the plasma (see Supplementary Fig. S4). The voltage and current waveforms are provided in Supplementary Fig. S1. Optical emission spectrum of this plasma is provided in Supplementary Fig. S2.

### Antibacterial efficacy of plasma on agar plates

Approximately ~1.6×10^6^ CFU of cells in late logarithmic growth phase were plated to determine the inhibition zone, which consisted of the region where bacterial growth was inhibited after incubation at 37 °C overnight.

### Measuring the level of DNA fragmentation

The DNA damage diffusion (DDD) Assay allows the detection of dsDNA breaks in single bacterial cells^28^. This assay was modified to quantify the level of dsDNA breaks in plasma treated, single bacterial cells *in situ* immediately after exposure.

Briefly, cells in late logarithmic phase were washed and diluted in PBS to OD_600_ = 0.07 (~2.3 × 10^7 ^CFU/mL), spread on plates and from there transferred to agarose-coated slides. This was exposed to the plasma, after which samples were immediately covered with 0.65% low-melting-point agarose (Sigma). After setting at 4 °C, slides were incubated for 5 minutes in lysis solution (2% (w/v) SDS, 0.05 M EDTA, 0.1 M dithiothreitol, pH 11.5), rinsed with distilled water, dehydrated with ice-cold ethanol for 3 minutes, and dried at 80 °C. For *S. aureus,* cells were incubated with lysostaphin (0.05 mg/ml) and lysozyme (0.25 mg/ml) for 15 minutes at 37 °C prior to the lysis step. Samples were stained with SYBR Gold (Invitrogen) for 5 minutes and analysed with fluorescence microscopy (Zeiss) with a 100x objective and GFP filter.

Images were collected with a high resolution monochromatic camera (AxioCam HRm, Zeiss), using the Axiovision Release 4.8.1 software. Images of single bacteria were collected at the treatment site (d = 0) and at 10, 20 and 30 mm from this site (Fig. 1A). The treatment site was identified as the location immediately below the centre of the visible plasma jet.

Intact genomic DNA from single cells appeared as a bright, condensed centre and a peripheral region of DNA loops (“core”). DNA with double stranded breaks presented DNA fragments dispersed around the core (“halo”). The level of DNA fragmentation was quantified by calculating the radius of the halo using the following algorithm implemented in Wolfram Mathematica 10.0 1.0 (Wolfram Research Inc.). The mean pixel brightness *B* as a function of the radius *r* from the centre of the halo can be modelled by the expression 
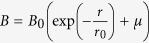
. where *B*_*0*_ denotes a brightness scale; *μ* denotes the background noise (normalised to *B*_*0*_); *r*_*0*_ denotes the halo size (i.e. the halo intensity decay scalelength). This technique provides an objective analysis of the results, subtracts background noise and is independent of the absolute level of illumination. Results are presented as the ratio of the radius of each plasma-treated cell divided by the mean of the radius of untreated cells, expressed as log_e_.

### Assessing the contribution of LTP-dependent UV radiation

A MgF_2_ window with transmittance ≥55% at 121 nm (Crystran Ltd.) was fitted to the lid of a Petri dish to create a sealed chamber containing the samples. UV radiation with wavelengths below 180 nm are absorbed in air^29^, and here cannot contribute to effects of LTP exposure. The air inside the chamber was replaced with He + 0.5 vol% O_2_ to ensure UV transmittance. Upon plasma treatment, samples were exposed to LTP-generated UV radiation, but not RNOS or charged species^30^. Samples were analysed as described.

### Statistical Analysis

One-way ANOVA followed by Tukey’s multiple comparisons test was performed using Prism v. 4.00 (GraphPad Software). Statistical significance was set at P < 0.05.

## Results

### Identifying experimental parameters based on the bacterial growth inhibition zone

In this study, *S.* Typhimurium was used as a model system for Gram-negative bacteria. Standard conditions for feed gas composition, treatment time, and the distance between sample and nozzle, were identified based on the microbial growth inhibition zone and considering sample temperature, for both the AP-DBD jet and μAPPJ (see Supplementary Figs S5 and S6, respectively). Based on the results, the feed gas was set at He with 0.5 vol% O_2_ and exposure at 90 second. Increasing the distance from nozzle to sample substantially decreased the bactericidal activity of the AP-DBD plasma jet (see Supplementary Fig. S5), but this was less evident with the μAPPJ (see Supplementary Fig. S6). Therefore, the sample distance was set at 30 mm and 5 mm distances, respectively. With these standardized conditions, the temperature of the AP-DBD plasma jet remained below 30 °C at the surface of the sample (see Supplementary Fig. S3). The AP-DBD plasma jet powered by a purely sinusoidal voltage (containing charged and neutral species) produced an inhibition zone of 25 mm radius in LB plates, whereas treatment with the μAPPJ yielded inhibition zones of 15–20 mm radius (Fig. 2B,D). No inhibition zone was seen upon exposure to feed gas only (Fig. 2F; for RF, data not shown), nor when LB plates were pre-treated with either LTP source prior to plating the cultures (see Supplementary Fig. S7). The latter result indicates that no long-lived, inhibitory secondary reactive species were produced upon interaction of plasma and the nutrients in the growth medium.

### LTP induced dsDNA breaks in a radially-dependent manner

To determine the role of the radial distribution of RNOS in LTPs, the induction of dsDNA breaks in *S.* Typhimurium induced by LTP treatments was assessed at the single cell level as a function of distance to the treatment site. This was made possible using our modified DDD assay in which bacteria are fixed in position in agarose, treated with LTP, and the integrity of the DNA per cell is analysed *in situ* immediately after the bactericidal treatment. This method retains the positional information. The radius of diffusion of DNA fragments through the agarose, which is size-dependent and originating from a single cell, provides the quantifiable readout of LTP-induced dsDNA breaks (see Methods) (Fig. 1B,C).

In *S.* Typhimurium treated with the AP-DBD plasma jet, the levels of DNA fragmentation at the treatment site and at 10 mm did not show a statistically significant difference (P < 0.05). However, these levels were significantly higher than the levels of DNA fragmentation observed at 20 mm (P < 0.01) and 30 mm from the treatment site (P < 0.0001) (Fig. 2A). Comparing this to the growth inhibition zone for cells on agar plates, 10 mm distance from the treatment site corresponded to 40% of the inhibition zone (Fig. 2B). No dsDNA fragmentation in plasma-treated bacteria was detectable beyond 10 mm, yet bactericidal action is still observed beyond that point. This trend was also observed when treating the Gram-positive bacterium *S. aureus* with an AP-DBD plasma jet (see Supplementary Fig. S8). In contrast, treatment with the μAPPJ resulted in detectable dsDNA breaks only in cells located at the treatment site (P < 0.0001) (Fig. 2C), and not at distances beyond this point (P > 0.05). Controls that were treated with the feed gas only (2 slm He + 0.5 vol% O_2_) at 5 mm from the nozzle with the AP-DBD plasma jet did not present detectable dsDNA breaks (P > 0.05) (Fig. 2E).

These results demonstrate that the levels of dsDNA damage in single bacterial cells induced by the AP-DBD plasma jet and the μAPPJ *S.* Typhimurium were inversely correlated to the distance to the treatment site.

### Direct interaction of plasma generated UV photons with the cellular DNA has an insignificant impact on DNA integrity

UV photons are produced in low-temperature atmospheric-pressure plasmas in addition to heavy particles, and can propagate unabsorbed through the feed gas used to generate plasma. UV is also a DNA damaging agent. To assess the contribution of LTP-generated UV photons to the bactericidal action and dsDNA fragmentation induced by LTPs, bacterial samples were exposed to LTP-generated UV radiation only, by blocking access of the plasma generated RNOS to the samples with a MgF_2_ window (see methods). For both plasma sources, exposure for 90 seconds to only the generated UV radiation was insufficient to cause a growth inhibition zone on agar plates (Fig. 3B,D). Importantly, no dsDNA fragmentation was detected at the treatment site in the bacterial cells exposed to the UV radiation generated by either plasma source (P > 0.05; Fig. 3A,C).

To compensate for the UV transmittance of the MgF_2_ window (≥55% at 121 nm) cells were also only exposed for 180 seconds to the UV generated by the μAPPJ plasma jet. This also did not result in growth inhibition (Fig. 3F). Compared to that at in cells located at 1 cm and 2 cm away, a very low level of ds DNA fragmentation was detected at the treatment site (0 cm) (P < 0.05), Fig. 3E). However, this low level was not statistically significant different from that at the treatment site after 90 seconds of UV only exposure (Fig. 3C). Importantly, the full plasma (90s) exposure causes highly significant more DNA fragmentation than exposure to UV only (180s) at this position (0 cm) (****P < 0.0001; Fig. 2C). Thus, we conclude that direct interaction of LTP-generated UV with the treated cells is not a significant contributor to the overall amount of the observed dsDNA fragmentation.

## Discussion

LTPs have proved to be effective against several bacterial species in liquid suspensions, biofilms, fresh produce and medical equipment^4^,^31^. The bactericidal properties of LTPs have been ascribed mainly to reactive nitrogen and oxygen species, collectively referred to as RNOS. The density of these species can vary in the plasma jet^12^,^13^,^32^, which could result in spatial variability of LTP bactericidal efficacy and of mechanisms of activity. Here we show that the level of dsDNA damage in Gram-positive and Gram-negative bacterial cells indeed varies with a radial dependency independently of the plasma source used, and that this most likely is a result of RNOS spatial distribution. This may be of particularly relevance for the development of biomedical LTPs.

Our results showed that there is an inverse relationship between distance from the centre of the treatment site and the level of dsDNA breaks occurring to dsDNA contained in either Gram-positive or Gram-negative bacterial cells, and that this is induced by different types of plasma. The DNA damage we assess is determined immediately after plasma treatment and the levels are therefore independent of DNA repair mechanisms. Furthermore, the approach eliminates effects from plasma-liquid interactions.

Our findings on spatial variation of DNA damage in living cells are consistent with previous reports where a similar correlation was established for purified DNA^14^,^15^. Furthermore, we show that plasma-generated UV by both LTP sources used here is not a major contributing factor (Fig. 3), supporting the conclusion that RNOS are the main agents leading to DNA fragmentation in the cells^33^. This indicates a consistent trend with variable LTP plasmas, for which absolute levels of damage and exact radial variation will vary as shown in this comparative study.

If RNOS are indeed the major agents leading to DNA damage, then our finding of radial variation of DNA breakage in plasma-exposed cells is consistent with previous findings on the distribution and chemistry of damaging oxygen species. The concentration of RNOS may decrease with increasing radial distance from the core of the plasma and treatment site (radial decrease) as well as from the nozzle toward the treatment site at 0 cm (axial decrease) due to diffusion and advection. This has been directly shown for hydroxyl radical distribution in an atmospheric-pressure plasma jet^34^, and for atomic oxygen in a different setup^12^,^13^. In addition, changes in concentration in the axial direction have been described for some species including ozone generated by a μAPPJ plasma jet^32^,^35^. Radial variation of RNOS thus is consistent with our conclusion of RNOS contributing to the spatial variation of cellular dsDNA fragmentation. Spatial variation may therefore also be expected for RNOS-dependent damage to other essential biomolecules like proteins, lipids and carbohydrates^36^.

Treating a living cell as done here leads to complexity to identify the mechanism(s) causing the bactericidal activity. Multiple chemical reactions occur between the RNOS generated in the plasma and cell components, including reactive oxygen species that can directly interact with lipids and proteins at the cell’s surface^37^. Furthermore, uncharged reactive species in the plasma such as hydrogen peroxide^38^ and ozone^39^ can diffuse across the cell membrane and cause direct damage to intracellular components. Inside the cell, these uncharged species can also lead to the formation of other reactive and damaging radical species such as hydroxyl and superoxide radicals that may cause damage.

Other RNOS besides ozone and hydrogen peroxide that are delivered by the plasma may also contribute to dsDNA breakage and bactericidal activity, including the uncharged, highly reactive and long-lived singlet delta oxygen and both charged and uncharged reactive nitrogen species^21^,^40^. As yet, no information in the literature is available on the spatial distribution of these species in and beyond the plasma jet.

Our data indicate that two other, non-RNOS sources of damage did not contribute to the observed bactericidal effect or DNA fragmentation. A self-induced electric field, as present in an AP-DBD plasma could have contributed by causing membrane damage^41^. However, our results obtained with the μAPPJ – where the electric field and charged particles are restricted to the core and are not present in the effluent region of the plasma – argue against a significant role of an electric field, since the levels of dsDNA damage directly under the nozzle are comparable for both plasma jets (Fig. 2). Furthermore, our data indicate that the direct contribution of plasma-generated UV to the bactericidal effect and DNA fragmentation is negligible. This is in agreement with results derived from a different LTP source^42^. This does not exclude a possible contribution from UV radiation generated by the plasma in the formation of reactive oxygen species in the gas phase^43^. Taken together, our data support the conclusion that the RNOS generated by both types of plasmas are key to eliciting the observed bactericidal effect and dsDNA fragmentation in the cells (Fig. 2).

Bactericidal activity was evident beyond the region with detectable DNA breakage. In these regions, the direct interaction of RNOS with macromolecules such as peptidoglycans, lipids and proteins in the cell wall and plasma membrane, respectively, could dominate the redox reactions leading to cell death^44^,^45^. Different types of macromolecular damage and induction of stress responses, including to ROS, occur as a result of plasma exposure, as shown at population level^46^. dsDNA breaks below the detection limit of the DDD assay and oxidation of nucleotide bases may also contribute to the bactericidal activity at these sites further from the plasma core. Assessing spatial variation using the single cell level approach, of a broad range of cellular damages, and with a high sensitivity may enhance our understanding of the range of molecular mechanisms that lead to plasma-induced bactericidal effects. Our future aim is to establish a tight correlation between the composition of the plasma, the distribution of the reactive species, and the nature of the biological damage. This can then inform optimal plasma design for specific applications.

Clinical trials on LTP for wound healing, have shown to reduce but not eliminate bacterial loads^47^,^48^. As our study shows, one of the biomolecules targeted by LTPs is the bacterial DNA. Double stranded DNA breaks will be lethal if unrepaired. However, cells with sub-lethal DNA damage that recover from LTP treatment may develop advantageous mutations for their survival. Our results demonstrate that variable DNA damage may occur during LTP therapy in bacteria in regard to the position of a cell in the plasma jet. Therefore, future work on developing LTP for clinical therapy may need to consider that bacterial mutations may arise at sites and under conditions, like those in the clinical study, where a subset of the bacterial population survives the LTP treatment.

Most reports on the bactericidal effect of plasma present the average effects on a treated population of cells, and do not consider possible heterogeneity of effects within that population^10^,^49^,^50^. Conversely, active spectroscopic plasma studies can provide information on the spatial distribution of RNOS, but are dissociated from the mechanisms for the induction of DNA damage and bacterial elimination^12^,^13^,^32^,^35^. Our novel quantitative, single cell DNA damage diffusion assay proved to be a robust and direct tool that retains spatial information on DNA damage induced by LTP in single bacterial cells, both Gram-positive and Gram-negative. Using this approach, we identified a radial dependence of plasma-induced dsDNA damage in the context of living cells. This provides new insight into the biological action of LTPs and demonstrates the importance of the radial distribution of plasma-induced particles in eliciting specific bactericidal effects. In the future, our approach could be combined with further analyses of the composition and spatial distribution of RNOS in the plasma to determine their contribution to bacterial inactivation.

Low-temperature plasma is a promising multi-target antibacterial therapy, and applied separately or in a combinatorial therapy^47^ this could be an effective topical therapy, for example for treating infected wounds. Ongoing research is increasing our understanding of the physical properties of plasmas and engineering of plasma delivery with the aim to generate plasmas that are both defined and controlled^51^,^52^. The results described here show the importance of the radial distribution of plasma-induced particles in the bactericidal properties of plasma. Thus continuing to integrate the plasma physics with biological analyses will be crucial towards developing plasma therapies to the point where this can be delivered effectively, controlled and safely to a biological sample.

## Additional Information

**How to cite this article**: Privat-Maldonado, A. *et al*. Spatial Dependence of DNA Damage in Bacteria due to Low-Temperature Plasma Application as Assessed at the Single Cell Level. *Sci. Rep.* **6**, 35646; doi: 10.1038/srep35646 (2016).

## Supplementary Material

Supplementary Information
