# Supplementary material for: Spatial Dependence of DNA Damage in Bacteria due to Low-Temperature Plasma Application as Assessed at the Single Cell Level
Source: Sci Rep. 2016 Oct 19;6:35646. doi: 10.1038/srep35646 (PMC5069486; doi:10.1038/srep35646)
Supplement: Supplementary Information [file srep35646-s1.pdf]

Supplementary information

Spatial Dependence of DNA Damage in Bacteria due to Low-Temperature Plasma Application as Assessed at the Single Cell Level

Angela Privat-Maldonado[1,2], Deborah O'Connell[2], Emma Welch[1], Roddy Vann[2], Marjan W. van der Woude[1,*]

Affiliations:
[1] Centre for Immunology and Infection, Department of Biology and Hull York Medical School, University of York, York, U.K.
[2] York Plasma Institute, Department of Physics, University of York, York, U.K.

Raw data underpinning the figures in the main manuscript and Supplemental information file, where relevant, is available online at
doi 10.15124/5a031284-377f-4020-87ac-c8ab21218d6e

Privat-Maldonado et al, Fig. S1

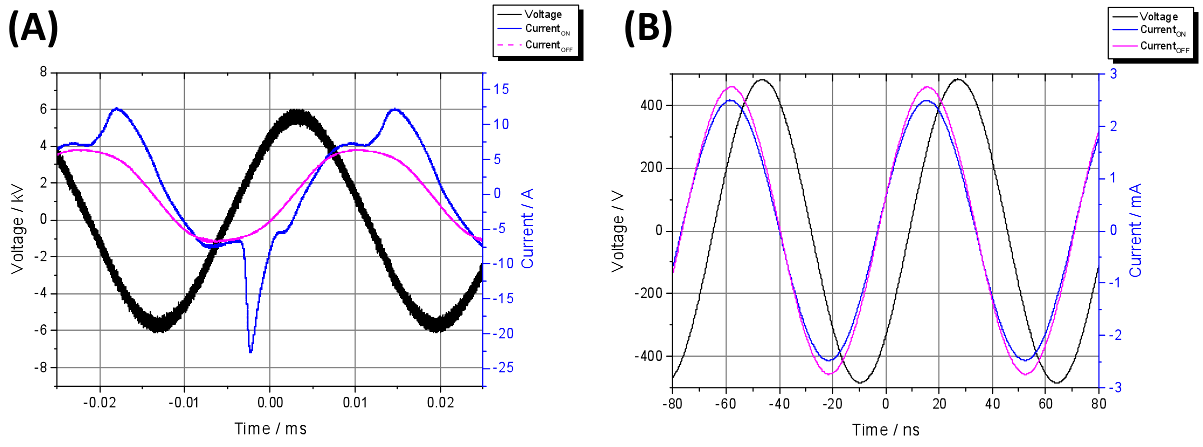

Figure S1. ***Representative voltage and current waveforms of both LTPs.*** The voltage (black) and current for plasma on (blue) and plasma off (magenta) were measured in **(A)** the sinusoidally-driven AP-DBD plasma, measured with a high voltage probe (Model P6015A, Tektronix) and a current monitor (Model CM-100-L, Ion Physics Corporation); and **(B)** the µAPPJ, where voltage and current were measured using the Vigilant Power Monitor (SOLAYL SAS). V: volts,; kV: kilovolts. Current expressed in Amperes (A), solid line: current plasma on; dashed line: current plasma off. Time expressed in milliseconds (ms) and nanoseconds (ns).

Privat-Maldonado et al, Fig. S2

(A)

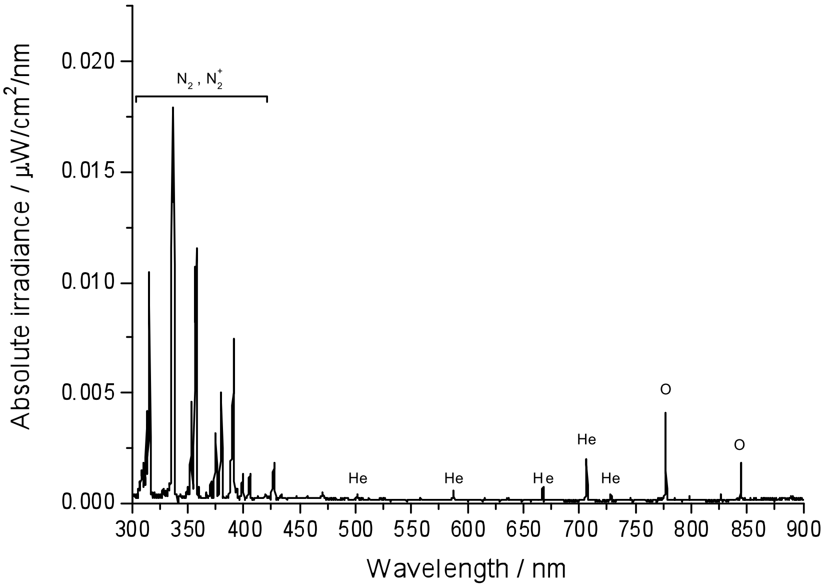

(B)

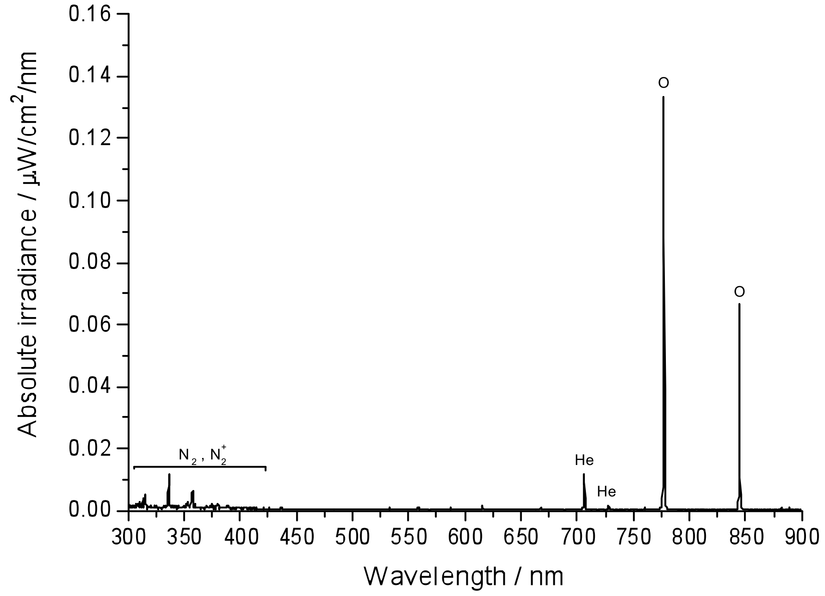

Figure S2. ***Representative optical emission spectra of both LTPs.*** **(A)** Optical spectrum of the AP-DBD plasma jet measured in the effluent region at 5 mm from the nozzle. Plasma generated with 2 slm He + 0.5 vol % O_2 and 12 kV, 31 kHz. **(B)** Optical spectrum of the µAPPJ measured between the electrodes, 2 mm above the nozzle. Plasma generated with 2 slm He + 0.5 vol % O_2, 13.56 MHz. Plots indicate the presence of helium (501 nm, 587 nm, 667 nm, 706 nm, and 728 nm), atomic oxygen (777 nm and 844 nm) and nitrogen molecular and ionic species between 300 and 435 nm. Spectra were obtained using the HR4000CG-UV-NIR spectrometer (Ocean Optics).

Privat-Maldonado et al, Fig. S3

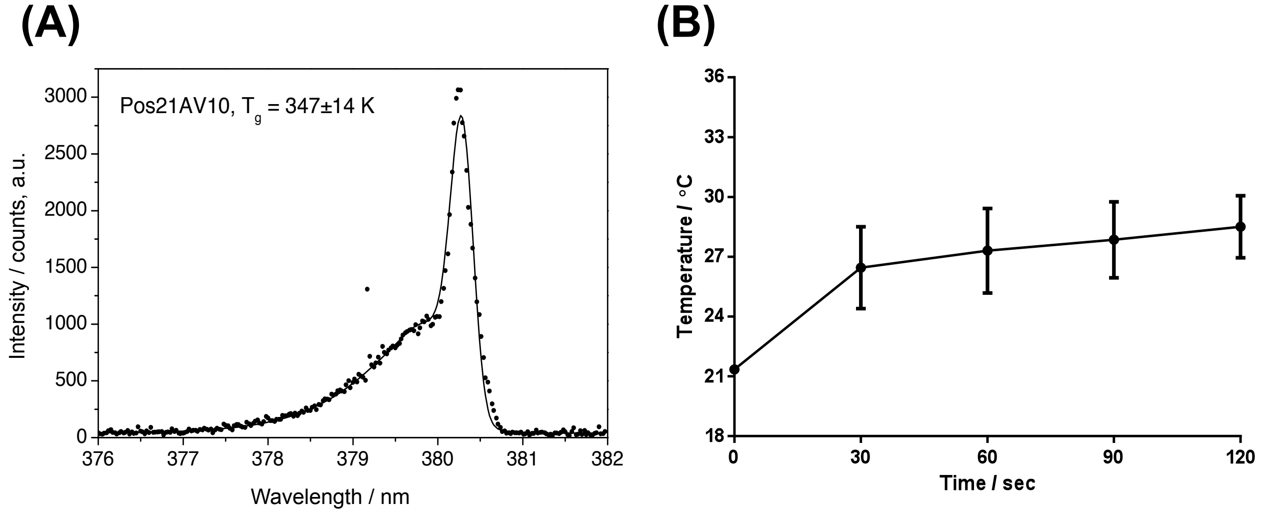

Figure S3. ***Gas temperature of the AP-DBD plasma jet.*** **(A)** The macroscopic temperature of the plasma jet in the visible fraction (at 21 mm from the nozzle) was calculated from the analysis of the second positive system of N_2 (see Methods). Spectra were analysed in the range between 376 – 382 nm. Plot represents the measured optical emission spectrum of N_2 for the transition between the electronic states C $^3\Pi_u$ and B $^3\Pi_g$. Solid line: best fit simulated spectrum; dots: experimental values. Temperature (T_g) expressed in degree Kelvin (74 ± 14 °C). **(B)** Temperature of the AP-DBD plasma jet at the surface of the sample (30 mm from the nozzle) at the treatment site measured with a thermocouple (K-probe, Tenma). The thermocouple was placed over an agar plate to determine the temperature the sample would experience during treatment. Measurements were taken every 30 seconds for 120 seconds. Temperature expressed in degree Celsius. Mean values plotted ± S.D. Time expressed in seconds (sec). **(A,B)** Plasma generated with 2 slm He + 0.5 vol % O_2.

Privat-Maldonado et al, Fig. S4

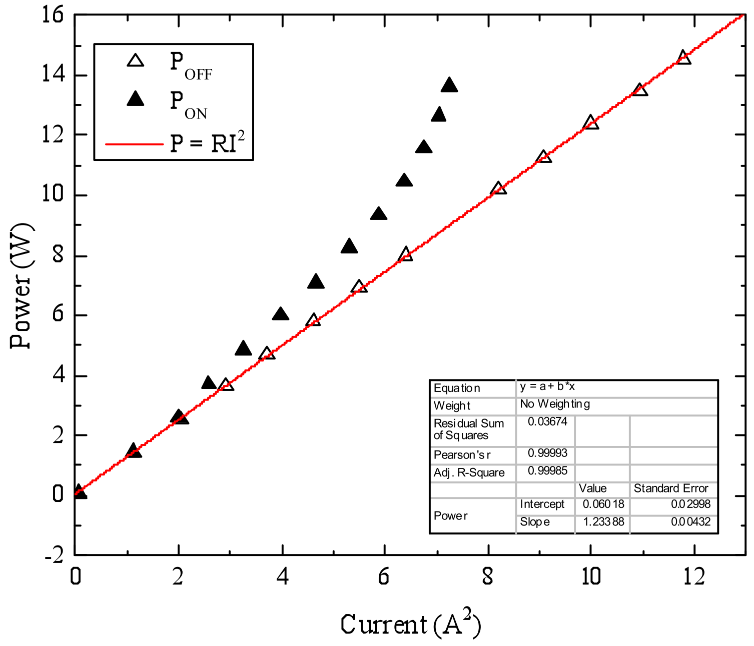

Figure S4. ***Representative dissipated power in the µAPPJ.*** The power was measured with the Vigilant Power Monitor (SOLAYL SAS) using the subtractive method (See Methods). The power measured with (P_{ON}) and without plasma (P_{OFF}) is shown as a function of the current. W: Watts. Current expressed in Amperes (A). Closed triangles: Power plasma on; open triangles: power plasma off.

Privat-Maldonado et al, Fig. S5

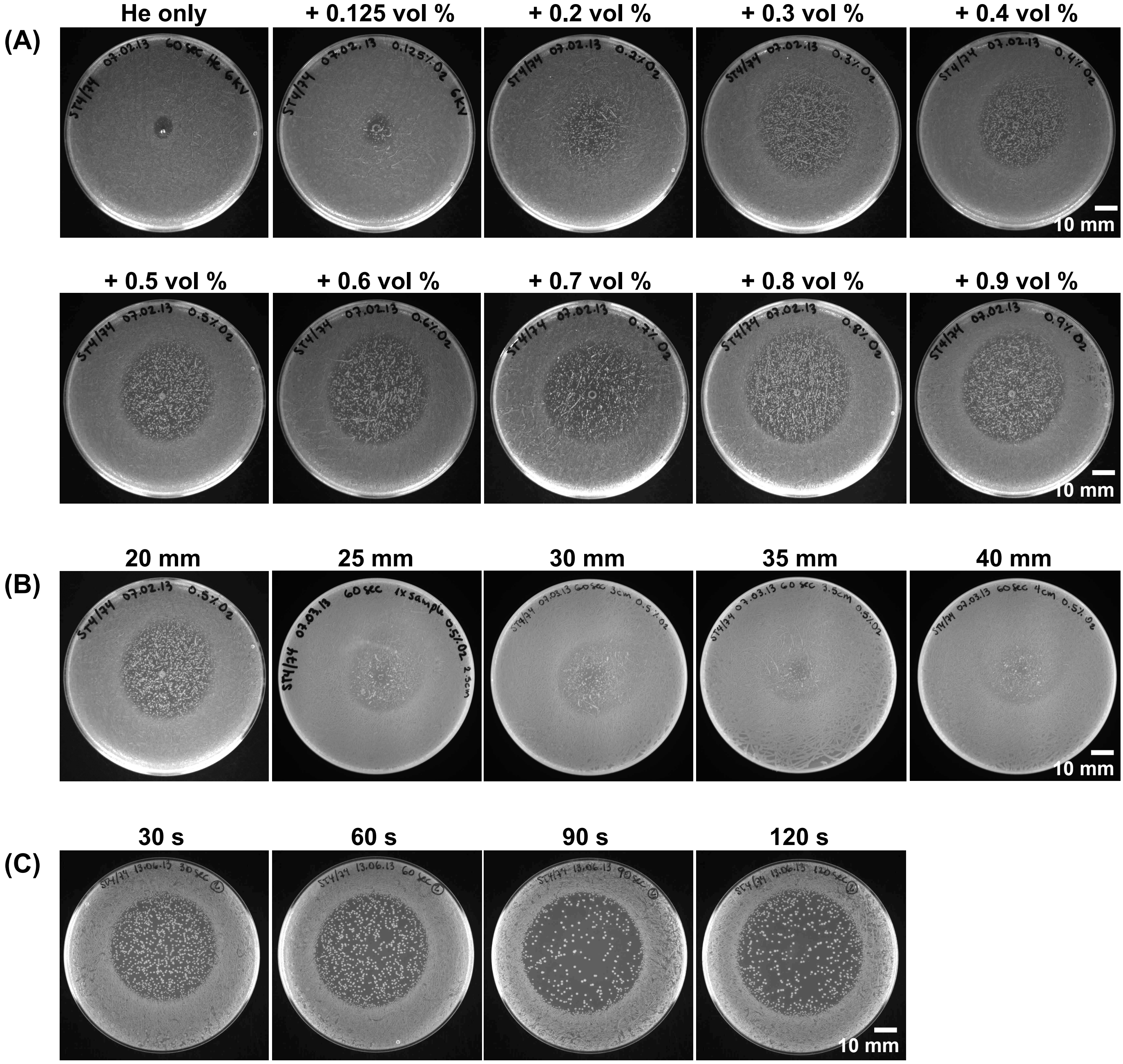

Figure S5. ***Standardization of AP-DBD plasma jet parameters for assessing S.*** **Typhimurium** ***growth inhibition.*** Representative plate showing growth inhibition of *S.* Typhimurium as a result of plasma treatment. Plasma was generated with gas flow at 2 slm, 6 kV and 123 kHz, with the following variable parameters: **(A)** Percentage of O_2 in the gas admixture varying from 0 to 0.9 vol % O_2, with 60 second treatment and 20 mm distance from nozzle to sample. **(B)** Varying distance from the nozzle to the sample at 20, 25, 30, 35 or 40 mm, with 0.5 vol % O_2 in gas admixture, and 60 seconds exposure. **(C)** Varying length of treatment of 30, 60, 90 or 120 seconds, using 0.5 vol % O_2 in the gas admixture, and at 30 mm distance from nozzle. **(A)** and **(B)** were performed using the single dielectric electrode configuration and **(C)** using the double electrode configuration for DBD as shown in Fig. 1. Scale bar 10 mm. Based on these results variable parameters were set at 0.5% vol O_2 in the gas admixture, 30 mm distance of nozzle to sample and 90 seconds exposure.

Privat-Maldonado et al, Fig. S6

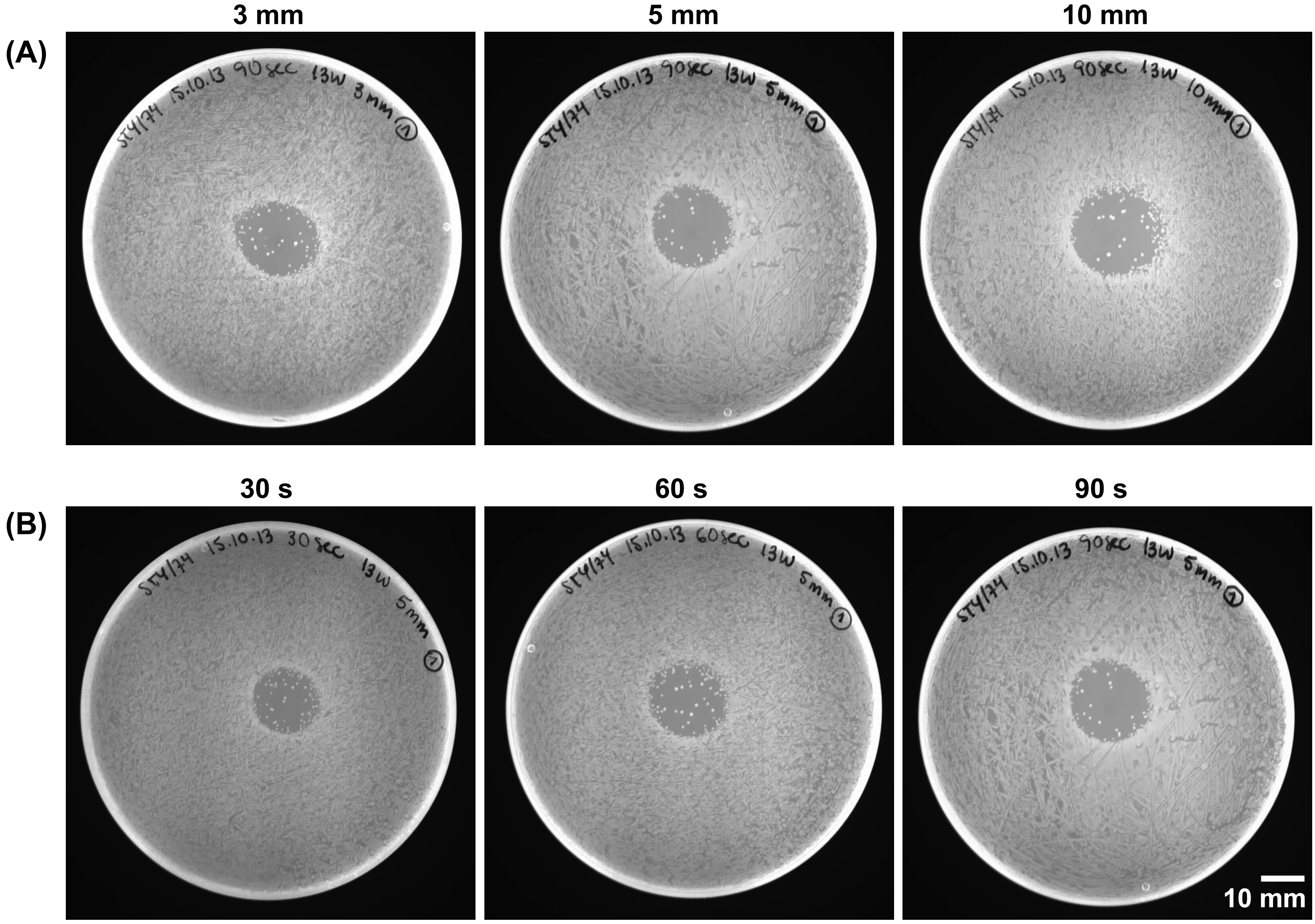

Figure S6. ***Standardization of µAPPJ plasma jet parameters for assessing S.*** **Typhimurium** ***growth inhibition.*** Representative plate showing growth inhibition of *S.* Typhimurium in plates exposed to µAPPJ jet **(A)** at 3, 5 and 10 mm distance from the nozzle for 90 seconds exposure and **(B)** at fixed distance of 5 mm for 30, 60 or 90 seconds exposure. Plasma was generated with 2 slm He + 0.5 vol % O_2, 13.56 MHz. Scale bar 10 mm.

Privat-Maldonado et al, Fig. S7

AP-DBD plasma jet

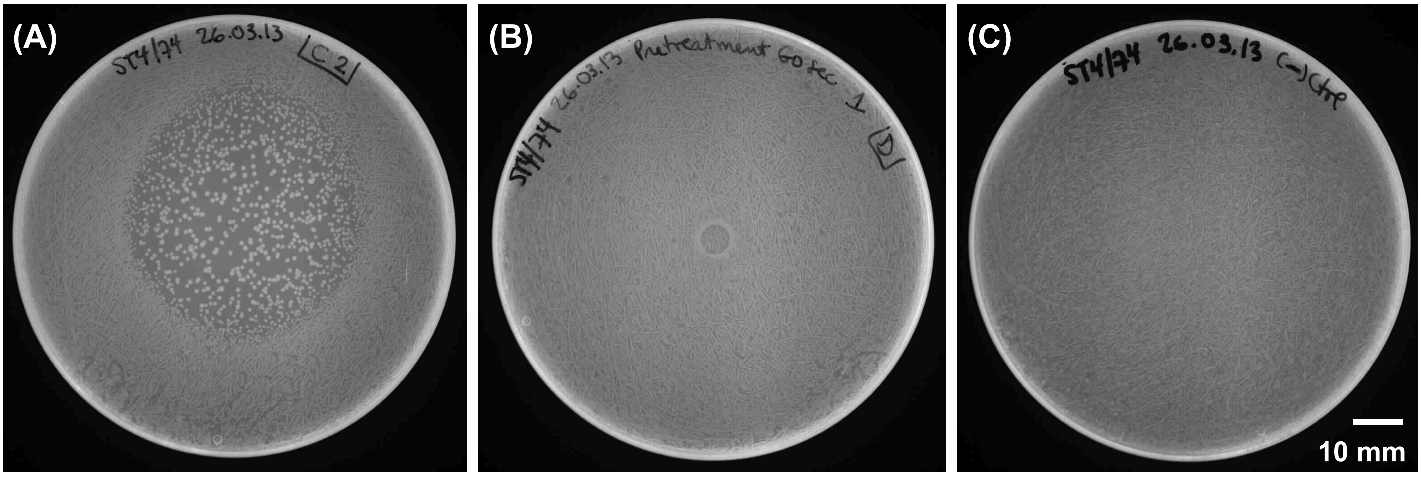

µAPPJ jet

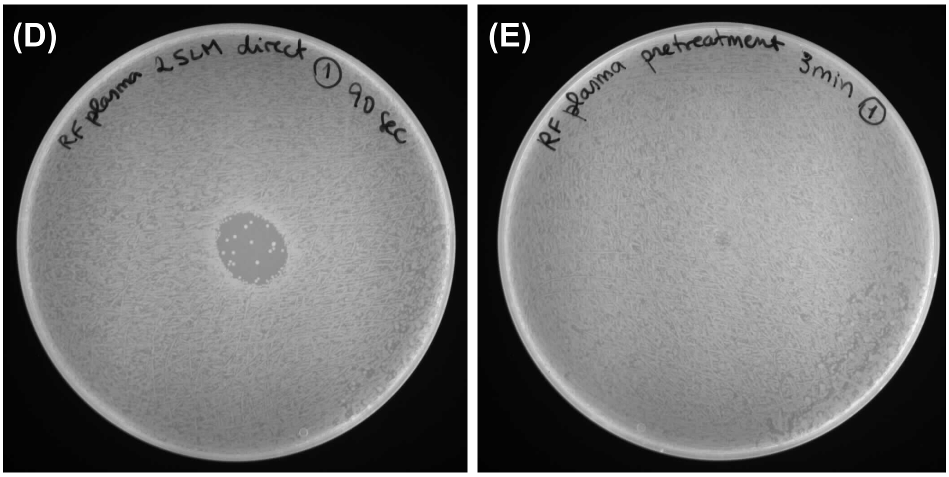

Figure S7. ***Pre-treatment of LB agar plates with either plasma jet did not affect bacterial growth.*** Representative plate showing growth inhibition of *S.* Typhimurium in plates exposed to AP-DBD plasma jet **(A)** for 30 seconds and **(B)** plates pre-treated for 60 seconds prior to spreading of bacteria. Plasma was generated in the AP-DBD plasma jet with 2 slm He + 0.5 vol % O_2, 6 kV, 123 kHz, 25 mm distance from nozzle to sample. **(C)** Untreated control. **(D**) Plates with *S.* Typhimurium exposed to µAPPJ jet for 90 seconds or **(E)** plates pre-treated with the µAPPJ jet for 180 seconds prior to spreading of bacteria. Plasma was generated in the µAPPJ jet with 2 slm He + 0.5 vol % O_2, 13.56 MHz, and 10 mm distance from nozzle to sample. Scale bar 10 mm. Pre-treated agar plates had a central indentation as a result of plasma exposure, but confluent bacterial growth was observed.

Privat-Maldonado et al, Fig. S8

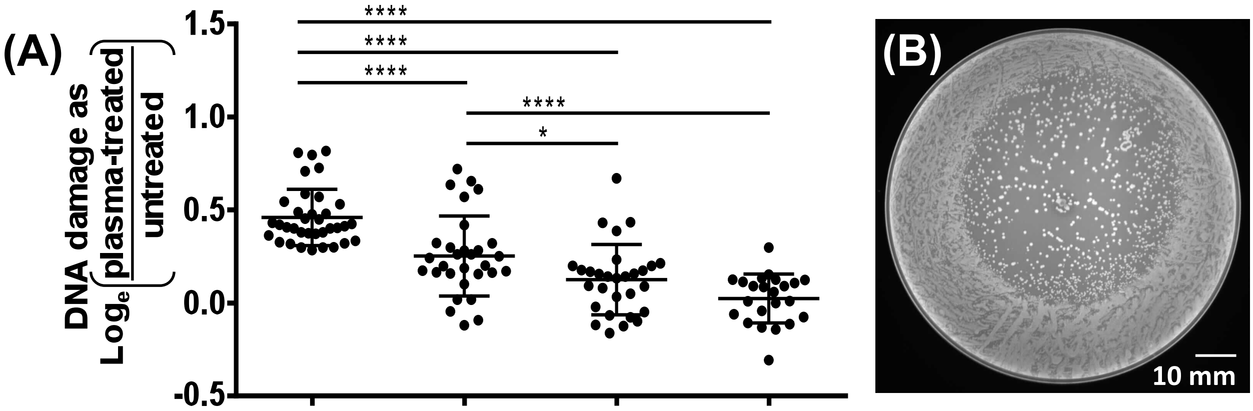

Distance to treatment site	0 cm	1 cm	2 cm	3 cm
% Radius of inhibition zone	0%	36%	71%	107%

Figure S8. ***Plasma treatment on S. aureus induced dsDNA breaks in a radial dependent manner.*** The Gram-positive *S. aureus* was exposed to the AP-DBD plasma jet operating at 12 kV and 31 kHz, with a gas flow of 2 slm and 0.5 vol % O_2 gas admixture. Bacteria were treated for 120 seconds. **(A)** Plot shows DNA damage on plasma-treated *S. aureus* at the single cell level as a function of their position to the treatment site (d = 0 cm). Each dot represents a single cell; horizontal bars: mean values ± S.D.; ****: P<0.0001; *: P<0.05. Ratio expressed as $\log_e$ ((radius plasma-treated cells)/(mean radius untreated cells)). **(B)** Representative plate showing growth inhibition of *S. aureus* as a result of plasma treatment. Scale bar 10 mm.
